# BCPA {*N*,*N*′-1,4-Butanediylbis[3-(2-chlorophenyl)acrylamide]} Inhibits Osteoclast Differentiation through Increased Retention of Peptidyl-Prolyl *cis-trans* Isomerase Never in Mitosis A-Interacting 1

**DOI:** 10.3390/ijms19113436

**Published:** 2018-11-01

**Authors:** Eugene Cho, Jin-Kyung Lee, Jee-Young Lee, Zhihao Chen, Sun-Hee Ahn, Nam Doo Kim, Min-Suk Kook, Sang Hyun Min, Byung-Ju Park, Tae-Hoon Lee

**Affiliations:** 1 Department of Oral Biochemistry, Dental Science Research Institute, School of Dentistry, Chonnam National University, Gwangju 61186, Korea; karu53@jnu.ac.kr (E.C.); sun3193@hotmail.com (S.-H.A.); bjpark@jnu.ac.kr (B.-J.P.); 2 Department of Molecular Medicine (BK21plus), Chonnam National University Graduate School, Gwangju 61186, Korea; wlsrud1945@naver.com (J.-K.L.); Chinaczhao@163.com (Z.C.); 3 New Drug Development Center, DGMIF, 80 Chumbok-ro, Dong-gu, Daegu 41061, Korea; jyoung@dgmif.re.kr (J.-Y.L.); shmin03@dgmif.re.kr (S.H.M.); 4 NDBio Therapeutics Inc., S24 Floor, Songdogwahak-ro 32, Yeonsu-gu, Incheon 21984, Korea; namdoo@ndbio.co.kr; 5 Department of Oral and Maxillofacial Surgery, School of Dentistry, Chonnam National University, Gwangju 61186, Korea; omskook@jnu.ac.kr

**Keywords:** Pin1, DC-STAMP, BCPA, osteoclast, osteoporosis

## Abstract

Osteoporosis is caused by an imbalance of osteoclast and osteoblast activities and it is characterized by enhanced osteoclast formation and function. Peptidyl-prolyl cis-trans isomerase never in mitosis A (NIMA)-interacting 1 (Pin1) is a key mediator of osteoclast cell-cell fusion via suppression of the dendritic cell-specific transmembrane protein (DC-STAMP). We found that *N*,*N*′-1,4-butanediylbis[3-(2-chlorophenyl)acrylamide] (BCPA) inhibited receptor activator of nuclear factor kappa-B ligand (RANKL)-induced osteoclastogenesis in a dose-dependent manner without cytotoxicity. In addition, BCPA attenuated the reduction of Pin1 protein during osteoclast differentiation without changing *Pin1* mRNA levels. BCPA repressed the expression of osteoclast-related genes, such as *DC-STAMP* and osteoclast-associated receptor (*OSCAR*), without altering the mRNA expression of nuclear factor of activated T cells (*NFATc1*) and cellular oncogene fos (*c-Fos*). Furthermore, Tartrate-resistant acid phosphatase (TRAP)-positive mononuclear cells were significantly decreased by BCPA treatment compared to treatment with the Pin1 inhibitor juglone. These data suggest that BCPA can inhibit osteoclastogenesis by regulating the expression of the DC-STAMP osteoclast fusion protein by attenuating Pin1 reduction. Therefore, BCPA may be used to treat osteoporosis.

## 1. Introduction

Bone tissue in the skeleton is continuously renewed through bone formation by osteoblasts and bone resorption by osteoclasts [1]. Osteoblasts and osteoclasts play crucial roles in the formation of the skeleton and regulation of bone mass. An imbalance between the formation and function of osteoblasts and osteoclasts can cause diseases, including osteoporosis [2,3]. Osteoclasts are multinucleated cells formed by the fusion of mononuclear osteoclast precursor cells of the monocyte/macrophage lineage. Subsequent activity depends on the enlargement of osteoclasts and is a major factor controlling bone resorption. Although inhibition of osteoclastogenesis does not reverse osteoporosis in elderly people who may have a reduced osteoblast-driven bone formation activity [4], osteoclast activity affects osteogenesis possibly by stimulating osteoblasts either through combined therapy with other drugs or cross-talk between osteoclasts and osteoblasts via the RANKL/RANK/osteoprogeterin triad [5].

Osteoporosis is characterized by low bone mass and results in the increased susceptibility to fracture due to increased bone resorption relative to bone formation. Although osteoporosis can occur at all ages, this degenerative bone disease typically occurs in elderly people [6]. The most common osteoporosis-related fractures occur in the hip, spine and wrist. Osteoporosis is a common age-related degenerative bone disease. In a previous report, postmenopausal women were found to be at high risk. It has been estimated that women and men over 50 years of age have a 50% and 20% risk, respectively, of fracture during their remaining lifetime [7]. Osteoporosis is associated with morbidity, mortality and increased socioeconomic costs [8].

Peptidyl-prolyl cis-trans isomerase never in mitosis A (NIMA)-interacting 1 (Pin1) belongs to the parvulin subfamily of PPIases. Pin1 specifically recognizes phosphorylated Pro-directed Ser/Thr motifs. The conformational changes that occur after phosphorylation have a profound effect on various cellular processes, including cell cycle and growth, cellular stresses, neuronal function, differentiation and survival and the immune response [9]. Pin1 enhances BMP and Wnt signaling and is involved in the regulation of Runx2 and Osterix, which are essential factors for osteoblast differentiation [10,11,12,13]. Fusion of osteoclasts is crucial for their function. Dendritic cell-specific transmembrane protein (DC-STAMP) is required for osteoclast fusion [14,15,16] and maintenance of bone mass [17]. Pin1 negatively regulates osteoclast fusion through DC-STAMP inhibition; as such, binding of Pin1 to DC-STAMP affects DC-STAMP localization [18]. Pin1 null mice reportedly exhibit low bone mass and bone mineral density [19]. In addition, osteoporotic disorders have been described in Pin1-deficient mice [20]. The collective findings indicate that Pin1 increases osteoblast formation and inhibits osteoclast fusion.

Increased expressions of cellular oncogene fos (c-Fos) and nuclear factor of activated T cells c 1 (NFATc1) by receptor activator of nuclear factor kappa B (RANK) signals are important in the regulation of osteoclastogenesis. c-Fos, a component of transcription factor activator protein-1 (AP-1), induces NFATc1 expression [21,22]. NFATc1 binds to the promoter region of DC-STAMP and induces its expression, which is crucial in the multinucleation process of osteoclasts [23,24]. 

The N-terminal WW domain of Pin1 functions in the recognition of phosphoproteins related to Pin1 regulation. Phosphorylation of a serine residue in the WW domain eradicates binding of Pin1 and its phosphoprotein [25]. Regulation of the WW domain is a potential treatment strategy for related diseases. In the present study, receptor-based in silico screening was used to find potential regulators of Pin1. *N*,*N*′-1,4-butanediylbis [3-(2-chlorophenyl)acrylamide] (BCPA) was identified as a novel Pin1 regulator that binds to the WW domain. Therefore, this study explored BCPA as a potential novel drug to regulate osteoclast activation by elucidating the effect and regulatory mechanism of BCPA on osteoclasts.

## 2. Results

### 2.1. Identification of BCPA by Receptor-Based in Silico Screening

**Figure 1:**
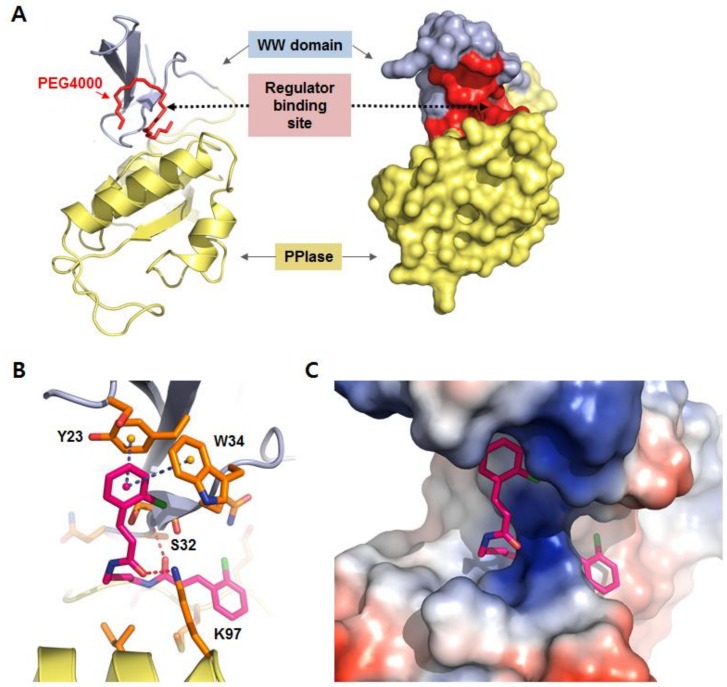
Binding model of BCPA and Pin1. (**A**) Three-dimensional structure of Pin1; the WW domain is depicted in blue, the PPIase domain in yellow and the regulator binding site in red. (**B**) Binding model of the BCPA and WW domain of Pin1. The red dashed lines indicate the hydrogen bonding interaction and the blue dashed lines indicate the pi-pi stacking interaction. (**C**) Surface model of BCPA on the WW domain binding site of Pin1.

The human Pin1 protein contains two domains, a hundred-residue prolyl *cis*/*trans* isomerase C-terminal catalytic domain and a small N-terminal called the WW domain. The WW domain is a critical recognition motif of Pin1, which recognizes Ser-Pro and Thr-Pro motifs [25]. The WW domain inhibition abolishes the capability of Pin1 to interact with phosphoproteins. The three-dimensional structure of the Pin1 WW domain comprises a triple β-parallel sheet. This structure is useful for receptor-based in silico screening. The WW domain forms a hydrophobic groove region at the boundary with the PPIase domain. A detergent, polyethylene glycol (PEG), binds to this region (Figure 1A) [26]. Based on the PEG binding structure, a docking study was performed to define the regulator binding site and to conduct the receptor-based in silico screening. The binding of PEG to Pin1 did not occur through chemical interactions and thus does not provide pharmacophore information but suggests possible binding site of other compounds in the same region. BCPA was singled out as a regulator of Pin1 with prospective activity. A proposed binding model of BCPA and Pin1 is depicted in Figure 1B. In the model, the p-chlorophenyl ring of BCPA forms an aromatic pi-pi stacking interaction with Y23 and W34 of the WW domain. At least 12 amino acids (L7, W11, S16, S18, Y23, Y24, N26, T29, S32, W34, Q35 and P37) are important for the binding of the ligand to the WW domain. Of these, Y23 and W34 are necessary for binding of the substrate peptide [27]. Hence, these pi-pi stacking interactions contribute to BCPA binding. Two hydrogen bonding interactions are critical for the binding between BCPA and the WW domain. The S32 residue on the WW domain and the K97 residue on the PPIase domain participate in hydrogen bonding. The WW domain is bound by PPIase domain and these two residues are known to make contacts through electrostatic interactions [27]. BCPA and Pin1 interacted in a very similar fashion with strong aromatic π–π stacking and two hydrogen bonds. The stable binding of BCPA at the WW domain binding site is depicted in Figure 1C.

### 2.2. BCPA Suppresses Osteoclast Differentiation without Cytotoxicity

**Figure 2:**
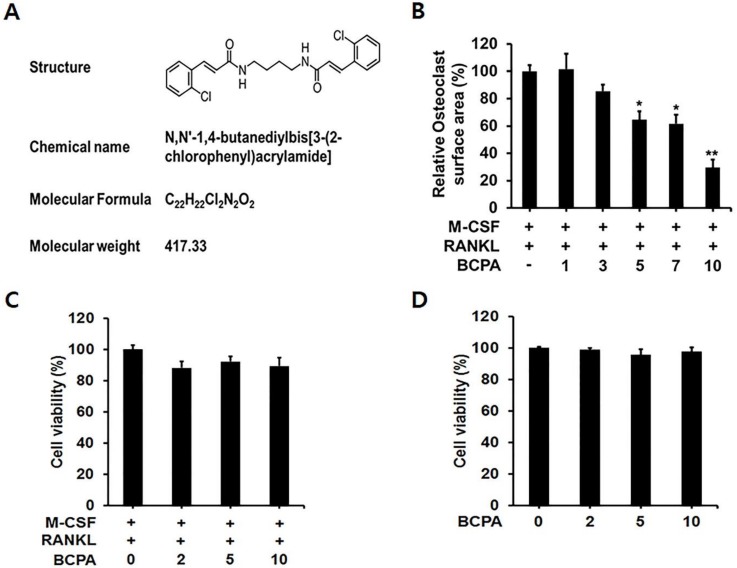
Effect of BCPA on osteoclast differentiation. (**A**) Chemical structure of BCPA. (**B**) Mouse bone marrow-derived macrophage cells were seeded in 96-well cell culture plates and treated with M-CSF (30 ng/mL), RANKL (50 ng/mL) and indicated concentrations of BCPA (μM) for 4 days. The cells were fixed and stained for TRAP to measure cell surface area. Mouse bone marrow-derived macrophages and MC3T3-E1 cells were seeded in a 96-well plate and treated with M-CSF (30 ng), RANKL (50 ng) and various concentrations of BCPA (0, 2, 5 and 10 μM) for 4 days (**C**) or 48 h (**D**). Cell viability was measured using the MTT assay. Data are presented as mean ± SD from three independent experiments. Data are presented as mean ± SD; * *p* < 0.05, ** *p* < 0.01.

To determine the effect of BCPA on osteoclast differentiation, TRAP staining was performed. BMMs were seeded in 96-well plates at 1 × 10^4^ cells per well. BCPA was added at the beginning of the culture with RANKL. BCPA reduced RANKL-induced osteoclast differentiation in a dose-dependent manner. Osteoclast differentiation was significantly reduced by BCPA at concentrations above 5 μM (Figure 2B). To determine whether the inhibitory effect of BCPA on osteoclast differentiation was due to cytotoxicity, the cytotoxicity of BCPA in osteoclasts was analyzed by the MTT assay. BCPA did not show cytotoxicity towards osteoclast differentiation for up to 4 days and was also not cytotoxic to MC3T3-E1 cells (Figure 2C,D). 

### 2.3. BCPA Inhibits Osteoclastogenesis

**Figure 3:**
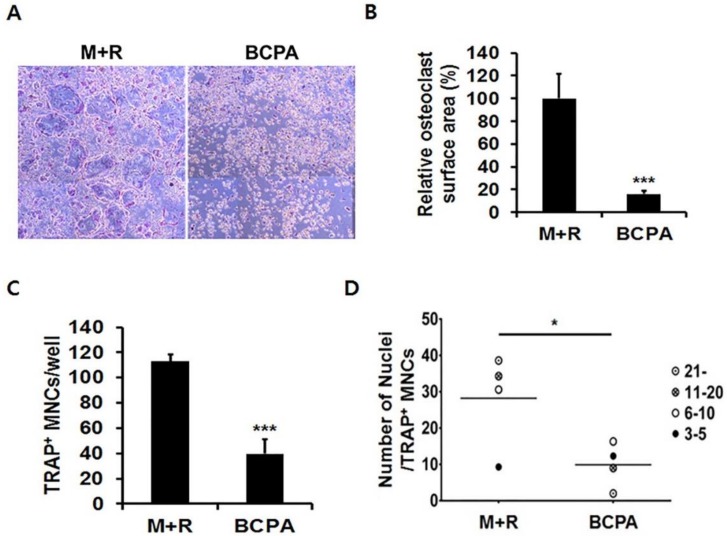
BCPA inhibits osteoclast differentiation. Mouse bone marrow-derived macrophage cells were seeded in 96-well plates and treated with M-CSF (30 ng/mL), RANKL (50 ng/mL) and BCPA (10 μM) for 4 days. To visualize osteoclasts, cells were fixed and stained with TRAP. (**A**) Stained cells were photographed using a 5× objective lens using a Leica DMIL LED microscope attached to a model DFC450C digital camera. (**B**) Surface area of TRAP-positive multinucleated cells was measured using Image J software. (**C**) Effect of BCPA on total number of TRAP-positive multinucleated cells. (**D**) In TRAP-positive multinucleated cells, cells containing three or more nuclei were counted. The distribution of nuclei in TRAP-positive multinucleated cells is depicted in a dot plot using GraphPad Prism 6. Data are presented as mean ± SD; * *p* < 0.05, *** *p* < 0.001.

To reaffirm the effect of BCPA in Figure 1B, TRAP staining was performed during RANKL-induced osteoclast differentiation. The formation of multinucleated giant cells was significantly reduced by BCPA (Figure 3A). In addition, the total numbers of TRAP-positive multinucleated cells and the surface area of osteoclasts were significantly reduced in the BCPA-treated group compared with the M-CSF- and RANKL-treated groups (Figure 3B,C). In particular, multinucleated cells possessing ≥6 nuclei were markedly decreased in TRAP-positive multinucleated cells (Figure 3D).

### 2.4. BCPA Regulates Osteoclastogenesis by Attenuating Pin1 Reduction

**Figure 4:**
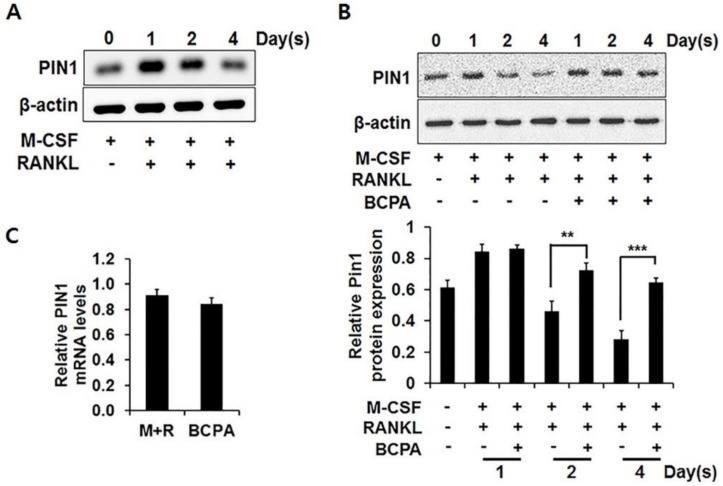
Regulation of Pin1 expression by BCPA. (**A**) Mouse bone marrow-derived macrophage cells were seeded in 6-well plates and treated with M-CSF (30 ng/mL), RANKL (50 ng/mL) and with/without (**B**) BCPA (10 μM) for 4 days. Whole cell protein was prepared and analyzed by western blot analysis using mouse monoclonal Pin1. Relative protein level of Pin1 was normalized to β-actin. Data are presented as mean ± SD; ** *p* < 0.01, *** *p* < 0.001. (**C**) Mouse bone marrow-derived macrophage cells were treated with M-CSF (30 ng/mL), RANKL (50 ng/mL) and BCPA (10 μM) and then cells were harvested after 4 days. (**C**) Total RNA was isolated using the TRIzol reagent. Gene expression was analyzed by quantitative real-time PCR.

To confirm whether BCPA regulates Pin1 because Pin1 inhibits osteoclast cell-cell fusion during osteoclastogenesis, western blot analysis was performed on osteoclast cells over time. Pin1 expression increased on day 1 and then decreased during osteoclast differentiation (Figure 4A). However, the decreased expression of Pin1 during osteoclast differentiation was attenuated by BCPA (Figure 4B). Next, to investigate whether BCPA mediated the changes in Pin1 expression, real-time PCR was performed. However, unlike the attenuation of the decrease in Pin1 protein expression, BCPA had no effect on Pin1 mRNA levels (Figure 4C). 

### 2.5. BCPA Inhibits Fusion of Osteoclasts by Reducing DC-STAMP

**Figure 5:**
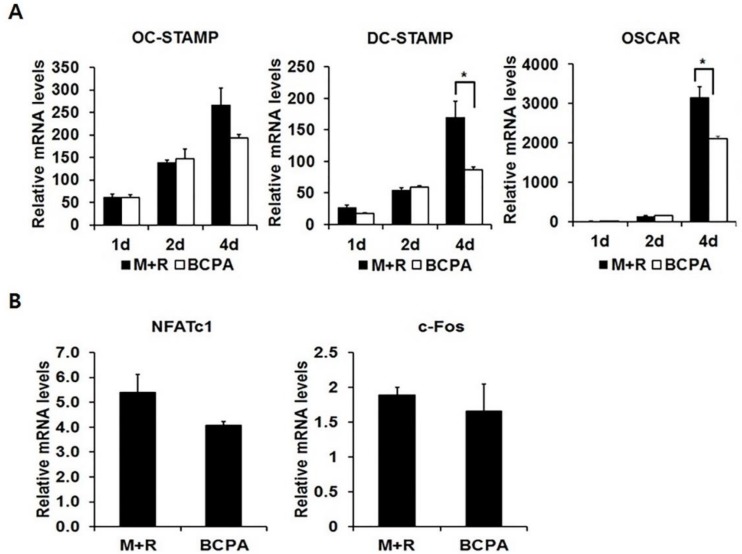
BCPA represses mRNA expression of *DC-STAMP* and *OSCAR*. (**A**) Mouse bone marrow-derived macrophage cells were seeded in 6-well plates and treated with M-CSF (30 ng/mL), RANKL (50 ng/mL) and BCPA (10 μM) for 4 days. (**B**) Mouse bone marrow-derived macrophage cells were treated with M-CSF (30 ng/mL), RANKL (50 ng/mL) and BCPA (10 μM) and then cells were harvested after 4 days. Total RNA was isolated using the TRIzol reagent. Gene expression was analyzed by quantitative real-time PCR. Data are presented as mean ± SD; * *p* < 0.05.

To examine the effect of BCPA on osteoclast-specific gene markers during osteoclastogenesis following RANKL stimulation, real-time PCR was performed. The mRNA levels of osteoclast marker genes *DC-STAMP* and *OSCAR* were significantly repressed by BCPA. However, the mRNA level of *OC-STAMP*, another gene that is essential for cell-cell fusion, was slightly but not significantly decreased (Figure 5A). During osteoclastogenesis, RANKL induces c-Fos expression, which leads to NFATc1 expression [21]. NFATc1 is a key transcription factor that regulates *DC-STAMP* and OSCAR expression [24,28]. Thus, the effect of BCPA on the mRNA expression of *NFATc1* and *c-Fos* was studied. BCPA did not have any evident effects (Figure 5B).

### 2.6. Pin1 Inhibition Enhances Osteoclastogenesis

**Figure 6:**
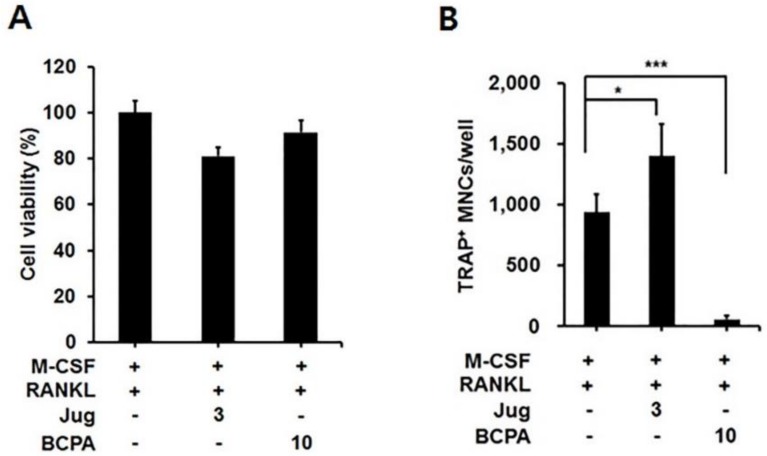
Effect of Pin1 inhibition during osteoclastogenesis. Mouse bone marrow-derived macrophage cells were seeded in 96-well plates and treated with M-CSF (30 ng/mL), RANKL (50 ng/mL), juglone and BCPA for 4 days. (**A**) Cell viability was measured by the MTT assay. (**B**) Effect of BCPA on the total number of TRAP-positive multinucleated cells.

To examine the function of Pin1 during osteoclast formation, Juglone known as Pin1 inhibitor was used as a negative control test group [29]. BCPA was slightly less cytotoxic than Juglone, even at high concentration (Figure 6A). In addition, osteoclast differentiation was significantly decreased by BCPA compared to the control but Juglone treatment induces osteoclast activation (Figure 6B).

## 3. Discussion

Osteoblasts and osteoclasts play a key role in the formation of the skeleton and regulation of bone mass. However, bone loss occurs due to imbalance of osteoblast and osteoclast activity, which causes osteoporosis and increases the risk of fracture [30,31]. Bisphosphates are the most commonly used antiresorptive drugs. They inhibit bone resorption but produce side effects that include reflux, esophagitis, gastritis and diarrhea [32,33]. Denosumab is a human monoclonal antibody to RANKL. It inhibits osteoclast differentiation but the side effects include cellulitis, flatulence and hypocalcemia [33]. Therefore, effective drugs for osteoporosis treatment and prevention of osteoclast differentiation and bone resorption are needed.

In this study, BCPA was selected as a molecule that bound to Pin1 through a library screening and we defined the binding site of BCPA in a docking study. Hydrogen bonding interactions with the S32 residue in the WW domain and K97 residue in the PPIase domain are critical for binding between BCPA. These results suggest that BCPA can bind to and regulate Pin1 (Figure 1). BCPA effectively inhibited RANKL-induced osteoclast differentiation in a dose-dependent manner and did not exhibit cytotoxicity at the effective concentration. In addition, the osteoclast surface area, number of TRAP-positive multinucleated cells and number of nuclei in TRAP-positive multinucleated cells were markedly decreased by BCPA (Figure 2 and Figure 3). These results suggest that BCPA can regulate osteoclast differentiation. Many previous studies demonstrated the genotoxicity of acrylamide [34,35]. The BCPA used in this study is also an acrylamide derivative. The International Agency for Cancer Research (IARC) has classified acrylamide as a human carcinogen (Group 2A). Exposure to acrylamide occurs through food, skin contact and respiration. Additionally, the exposure safety margin was calculated as BMDL_10_ 310 μg/kg (body weight/day) in the human safety standard set by JECFA. Based on in vitro experiments using osteoclasts and MC3T3-E1 cells, our results in Figure 2C,D showed that BCPA did not affect cell viability at concentrations 0–10 μM.

Pin1 enhances osteoblast differentiation through regulation of protein stability and transcriptional activity of the osteogenic transcriptional factor Runx2 [36]. Conversely, Pin1 inhibits osteoclast cell-cell fusion via DC-STAMP inhibition and Pin1-deficient or null mice display osteoporotic disorders that include low bone mass and bone mineral density [15,16,17]. Thus, it is assumed that BCPA may regulate Pin1 stabilization. In this study, the expression of Pin1 was decreased during osteoclast differentiation but this decrease was attenuated by BCPA. BCPA had no effect on Pin1 mRNA levels (Figure 4). 

BCPA markedly repressed the mRNA expression of DC-STAMP. The expression of DC-STAMP was increased in a time-dependent manner as opposed to decreased Pin1 expression during osteoclast differentiation (Figure 4A and Figure 5A). RANKL-induced osteoclast differentiation and DC-STAMP mRNA expression were sufficient for 4 days [37]. However, the expression of DC-STAMP decreased on day 4 but not on day 2, of differentiation, when Pin1 was not decreased by BCPA on day 4 (Figure 5A). These results suggest that maintenance of Pin1 expression by BCPA may affect osteoclast maturation, rather than osteoclast differentiation. In contrast, mRNA expressions of *c-Fos* and *NFATc1*, which are transcription factors regulating the expression of *DC-STAMP*, were not changed (Figure 5B). These results are consistent with the increased mRNA expression of *DC-STAMP* without changes in *c-Fos* and *NFATc1* expression in Pin1-deficient mice, suggesting that the reduced DC-STAMP mRNA expression by BCPA may be due to Pin1 [19]. DC-STAMP is a late-stage osteoclast marker. We performed western blot analysis several times to confirm the expression of DC-STAMP protein with anti-DC-STAMP antibody (MABF39-I, Merck Millipore, Burlington, MA, USA), which did not show reactivity with DC-STAMP in our experimental setup. We did not detect the DC-STAMP protein band and no specific commercial antibody is available for this experiment. In addition, BCPA significantly repressed the mRNA expression of *OSCAR* without altering *NFATc1* mRNA expression (Figure 5A), a transcription factor of *OSCAR* [28]. Furthermore, TRAP-positive multinucleated cells were significantly increased by the Pin1 inhibitor juglone (Figure 6). This result is also consistent with the increase in osteoclast numbers when treated with juglone or PIN1 small interfering RNA during RANKL-induced osteoclast differentiation of PDLCs and cementoblasts [38].

In conclusion, our results suggest that Pin1 reduction is attenuated by BCPA, thereby inhibiting osteoclasts fusions due to decreased DC-STAMP expression. Thus, BCPA acts as an inhibitor of osteoclast differentiation and has potential as a treatment of bone diseases, such as osteoporosis. However, further studies are required to understand how BCPA affects the stability of Pin1 and in vivo studies using animal models are needed to determine potential side effects and the effective concentration range of BCPA.

## 4. Materials and Methods

### 4.1. In Silico Screening of a Compound Library for Pin1 Regulators

In silico screening was performed by targeting the WW domain of Pin1 and the regulator binding site was modified based on several X-ray crystal structures of Pin1 bound to the detergent PEG4000 (3NTP.pdb and 3TDB.pdb) [39,40]. The Glide docking tool (Schrödinger LLC, New York, NY, USA), which uses a receptor grid based on the ligand binding site, was used to screen this library of 200,000 compounds (ChemBridge Corp., San Diego, CA, USA) [41]. The Glide tool filters molecules using XP (extra precision) modes [42]. The Glide score (docking score) and visual inspection were used to consider the candidate compound selection. The selection criteria for virtual screening hits were established using the Glide score. The Glide Score is a scoring function that separates compounds that do not bind to the protein and those that bind strongly after the docking simulation [42]. After Glide docking simulation, 200 compounds with the highest Glide score were shortlisted and the final candidates were selected by visual inspection. All 45 selected compounds were screened for their biological activities. Molecular graphics for the figures were generated by the PyMol molecular graphics package (http://www.pymol.org).

### 4.2. Materials

BCPA (*N*,*N*′-1,4-butanediylbis[3-(2-chlorophenyl)acrylamide]) was purchased from ChemBridge Corp. (Chembride ID, 7241340, San Diego, CA, USA). It was dissolved in dimethylsulfoxide (DMSO). Aliquots of the BCPA solution were stored at −20 °C. The stock solution of BCPA was diluted to the appropriate concentration before use.

### 4.3. Cell Culture

Mouse MC3T3-E1 cells were cultured in αMEM (Gibco, Grand Island, NY, USA) supplemented with 10% heat-inactivated fetal bovine serum (Hyclone, Logan, UT, USA), 100 U/mL penicillin and 100 μg/mL streptomycin (WELGENE, Seoul, Korea) and then maintained at 37 °C in a humidified incubator in an atmosphere of 5% CO_2_. 

### 4.4. Isolation and Differentiation of Mouse Bone Marrow-Derived Macrophages (BMMs)

Wild-type C57BL6/J mice purchased from Damul Science, Inc. (Daejeon, Korea) were housed in a specific pathogen-free facility following the guidelines in the Guide for the Care and Use of Laboratory Animals (Chonnam National University, Gwangju, Korea). Adult, male, 10-week-old C57BL/6J mice were used for this study. Collection of mouse primary mononuclear cells was approved by the IACUC at Chonnam National University (Approval No. CNU IACUC-YB-2017-70, 31 October 2017). After the hip, femur and tibia bones were isolated from C57BL6/J mice, all muscles that joined the bones were removed. All bones were crushed in a sterile mortar containing 5 mL αMEM and the supernatant was collected and filtered using a strainer. The isolated cells were seeded in a 100-mm culture dish and incubated for 1 day. Non-adherent cells were collected and centrifuged at 1200 rpm for 10 min at room temperature. BMMs were resuspended in culture medium containing 30 ng/mL of macrophage-colony stimulating factor (M-CSF) and incubated for 3 days. Cells were washed twice with Hanks Balanced Salt solution and added to the cell dissociation buffer. Collected cells were cultured with 30 ng/mL M-CSF for 1 day and then treated with 30 ng/mL M-CSF and 50 ng/mL RANKL for 1 to 4 days. 

### 4.5. MTT Assay

MC3T3-E1 and BMMs were seeded in 96-well plates at a density of 1 × 10^4^ cells per well. BMMs cells were treated with 30 ng/mL M-CSF and 50 ng/mL RANKL for the indicted culture time. MC3T3-E1 and MNCs were treated with BCPA at different concentration for 48 h and 4 days, respectively. The culture medium in each well was replaced with 20 μL of a 5 mg/mL stock solution of 3-(4,5-dimetylthiazol-2-yl)-2,5-diphenyltetrazolium bromide (MTT; Sigma-Aldrich, St. Louis, MO, USA). The cells were incubated for 1 h at 37 °C in a humidified incubator in an atmosphere of 5% CO_2_. After removal of the supernatants, the formazan crystals were dissolved in 100 μL of DMSO. The optical density was measured at 570 nm using a SPECTRA MAX i3x microplate reader (Molecular Devices, Sunnyvale, CA, USA). The optical density was first normalized to cell number (absorbance/cell number) and compared with that of the control (M-CSF- and RANKL-treated group).

### 4.6. Western Blot Analysis

BMMs were seeded in 6-well plates at a density of 4 × 10^5^ cells per well with 30 ng/mL M-CSF, 50 ng/mL RANKL and BCPA at the indicated concentrations. After 4 days, the cells were scraped for whole protein extraction. The proteins were electrophoretically separated by SDS-PAGE and transferred to polyvinylidene fluoride membranes (Millipore, Bedford, MA, USA). The membranes were blocked with Tris buffered saline-Tween (TBST) containing 5% skim milk for 1 h and incubated overnight at 4 °C with Pin1 (sc-46660; Santa Cruz Biotechnology, Santa Cruz, CA, USA), DC-STAMP (MABF39-I, Merck Millipore, Darmstadt, Germany) and β-actin (A5441; Sigma-Aldrich) primary antibodies. The membranes were washed four times for 5 min each time with TBST buffer and incubated with a secondary horseradish peroxidase-conjugated antibody (Santa Cruz Biotechnology). The reaction was detected using an Amersham ECL Western Blotting Detection Reagent kit (RPN2106; GE Healthcare, Chicago, IL, USA) and pictures were taken with an EZ-Capture MG device (ATTO, Tokyo, Japan).

### 4.7. Quantitative Real-Time PCR

BMMs were seeded in 6-well plates at a density of 4 × 10^5^ cells per well with 30 ng/mL M-CSF, 50 ng/mL RANKL and BCPA at the indicated concentrations. Total RNA was isolated from osteoclasts using TRIzol^®^ (Invitrogen, Carlsbad, CA, USA) according to the manufacturer’s protocol. Briefly, after removal of the culture medium, 500 μL of TRIzol^®^ reagent was added to each well. The cell extracts were vigorously mixed with 100 μL of chloroform and centrifuged at 13,000 rpm for 10 min at 4 °C. The aqueous phase was transferred to a new tube and mixed with 500 μL of isopropanol. The extracts were washed with 75% ethanol. The RNA pellet was dissolved in RNase-free water and the concentration was measured using the aforementioned SPECTRA MAX i3x microplate reader. First-strand cDNA was synthesized with 1 μg of RNA using the PrimeScript™ RT reagent kit (TaKaRa Bio, Shiga, Japan) according to the manufacturer’s protocol. Quantitative real-time PCR was performed using the Power SYBR^®^ Green PCR Master Mix (Life Technologies, Warrington, UK) on the QuantStudio^®^ 3 Real-Time PCR System (Applied Biosystems, Foster City, CA, USA). PCR was performed using the primers shown in Table 1. The PCR conditions were as follows: 95 °C for 10 min, 95 °C for 15 s and 60 °C for 1 min. The mRNA level was normalized to the level of glyceraldehyde-3-phosphate dehydrogenase (GAPDH) and compared with that of the control group. Gene expression was analyzed using the 2^−ΔΔ*C*t^ method.

### 4.8. Tartrate-Resistant Acid Phosphatase (TRAP) Staining

BMMs were seeded in 96-well plates at a density of 1 × 10^5^ cells per well with 30 ng/mL M-CSF and 50 ng/mL RANKL for 4 days. The cells were fixed in 3.8% paraformaldehyde for 30 min at room temperature and then stained for TRAP using an Acid Phosphatase, Leukocyte (TRAP) Kit (Cat. #387A, Sigma-Aldrich). The cells were incubated for 10~15 min at 37 °C under dark conditions and then washed twice with sterilized distilled water. Red-stained cells were considered differentiated osteoclasts and multinucleated cells contained three or more nuclei. Pictures were taken using a model DMIL LED microscope (Leica, Wetzlar, Germany).

### 4.9. Statistical Analysis

All data are expressed as the mean ± SD. Statistical analysis was performed using the unpaired two-tailed Student’s *t*-test with SPSS software version 22 (SPSS, Inc., Chicago, IL, USA). The difference between groups was considered statistically significant at *p* < 0.05. Graphs were plotted using GraphPad prism 6 (GraphPad Software Inc., La Jolla, CA, USA).

## Figures and Tables

**Table 1:** Primer sequences used for quantitative real-time PCR.

Gene	Sense Primer Antisense Primer	*T*_m_ (°C)	Accession Number
*mPin1*	5′-TTAATGGAAGGTGCGTAGGGT-3′	60	NM_023371
	5′-TTAATGGAAGGTGCGTAGGGT-3′		
*mc-Fos*	5′-CGAAGGGAACGGAATAAGATG-3′	55	NM_010234
	5′-GCTGCCAAAATAAACTCCAG-3′		
*mNFATc1*	5′-ACCACCTTTCCGCAACCA-3′	56	NM_016791
	5′-GGTACTGGCTTCTCTTCCGT-3′		
*mOC-STAMP*	5′-CAGAGTGACCACCTGAACAA-3′	56	NM_029021
	5′-TGCCTGAGGTCCCTGTGACT-3′		
*mDC-STAMP*	5′-GGGAGTCCTGCACCATATGG-3′	56	NM_029442
	5′-AGGCCAGTGCTGACTAGGAT-3′		
*mOSCAR*	5′-TCTGCCCCCTATGTGCTATC-3′	58	NM_175632
	5′-CAGCCCCAAACGGATGAG-3′		
*mGAPDH*	5′-TGTGTCCGTCGTGGATCTGA-3′	56	NM_001289726
	5′-GATGCCTGCTTCACCACCTT-3′		

## Abbreviations

BMMs	bone marrow-derived macrophages
MNCs	multinucleated giant cells
Pin1	peptidyl-prolyl cis-trans isomerase NIMA-interacting 1
BCPA	*N*,*N*′-1,4-butanediylbis[3-(2-chlorophenyl)acrylamide]
mRNA	messenger ribonucleic acid
PCR	polymerase chain reaction
DMSO	dimethyl sulfoxide

## References

[1] Chen X., Wang Z., Duan N., Zhu G., Schwarz E.M., Xie C. (2018). Osteoblast-osteoclast interactions. Connect. Tissue Res..

[2] Teitelbaum S.L. (2000). Bone resorption by osteoclasts. Science.

[3] Leibbrandt A., Penninger J.M. (2008). RANK/RANKL: Regulators of immune responses and bone physiology. Ann. N. Y. Acad. Sci..

[4] Weitzmann M.N. (2013). The Role of Inflammatory Cytokines, the RANKL/OPG Axis, and the Immunoskeletal Interface in Physiological Bone Turnover and Osteoporosis. Scientifica.

[5] Almeida M. (2012). Aging mechanisms in bone. Bonekey Rep..

[6] Hou Y.C., Wu C.C., Liao M.T., Shyu J.F., Hung C.F., Yen T.H., Lu C.L., Lu K.C. (2018). Role of nutritional vitamin D in osteoporosis treatment. Clin. Chim. Acta.

[7] Office of the Surgeon General (US) (2004). Bone Health and Osteoporosis: A Report of the Surgeon General. Rockv. (MD) Off. Surg. Gen..

[8] Harvey N., Dennison E., Cooper C. (2010). Osteoporosis: Impact on health and economics. Nat. Rev. Rheumatol..

[9] Lu K.P., Zhou X.Z. (2007). The prolyl isomerase PIN1: A pivotal new twist in phosphorylation signalling and disease. Nat. Rev. Mol. Cell Biol..

[10] Yoon W.J., Islam R., Cho Y.D., Woo K.M., Baek J.H., Uchida T., Van Komori Wijnen A., Stein J.L., Lian J.B., Stein G.S. (2013). Pin1-mediated Runx2 modification is critical for skeletal development. J. Cell. Physiol..

[11] Yoon W.J., Islam R., Cho Y.D., Ryu K.M., Shin H.R., Woo K.M., Baek J.H., Ryoo H.M. (2015). Pin1 plays a critical role as a molecular switch in canonical BMP signaling. J. Cell. Physiol..

[12] Lee S.H., Jeong H.M., Han Y., Cheong H., Kang B.Y., Lee K.Y. (2015). Prolyl isomerase Pin1 regulates the osteogenic activity of Osterix. Mol. Cell. Endocrinol..

[13] Shin H.R., Islam R., Yoon W.J., Lee T., Cho Y.D., Bae H.S., Kim B.S., Woo K.M., Baek J.H., Ryoo H.M. (2016). Pin1-mediated Modification Prolongs the Nuclear Retention of β-Catenin in Wnt3a-induced Osteoblast Differentiation. J. Biol. Chem..

[14] Yagi M., Miyamoto T., Sawatani Y., Iwamoto K., Hosogane N., Fujita N., Morita K., Ninomiya K., Suzuki T., Miyamoto K. (2005). DC-STAMP is essential for cell-cell fusion in osteoclasts and foreign body giant cells. J. Exp. Med..

[15] Chiu Y.H., Ritchlin C.T. (2016). DC-STAMP: A Key Regulator in Osteoclast Differentiation. J. Cell. Physiol..

[16] Maruyama K., Uematsu S., Kondo T., Takeuchi O., Martino M.M., Kawasaki T., Akira S. (2013). Strawberry notch homologue 2 regulates osteoclast fusion by enhancing the expression of DC-STAMP. J. Exp. Med..

[17] Fujita K., Iwasaki M., Ochi H., Fukuda T., Ma C., Miyamoto T., Takitani K., Negishi-Koga T., Sunamura S., Kodama T. (2012). Vitamin E decreases bone mass by stimulating osteoclast fusion. Nat. Med..

[18] Islam R., Bae H.S., Yoon W.J., Woo K.M., Baek J.H., Kim H.H., Uchida T., Ryoo H.M. (2014). Pin1 regulates osteoclast fusion through suppression of the master regulator of cell fusion DC-STAMP. J. Cell. Physiol..

[19] Shen Z.J., Hu J., Ali A., Pastor J., Shiizaki K., Blank R.D., Kuro-o M., Malter J.S. (2013). Pin1 null mice exhibit low bone mass and attenuation of BMP signaling. PLoS ONE.

[20] Islam R., Yoon W.J., Ryoo H.M. (2017). Pin1, the Master Orchestrator of Bone Cell Differentiation. J. Cell. Physiol..

[21] Ranger A.M., Gerstenfeld L.C., Wang J., Kon T., Bae H., Gravallese E.M., Glimcher M.J., Glimcher L.H. (2000). The nuclear factor of activated T cells (NFAT) transcription factor NFATp (NFATc2) is a repressor of chondrogenesis. J. Exp. Med..

[22] Gohda J., Akiyama T., Koga T., Takayanagi H., Tanaka S., Inoue J. (2005). RANK-mediated amplification of TRAF6 signaling leads to NFATc1 induction during osteoclastogenesis. EMBO J..

[23] Yagi M., Ninomiya K., Fujita N., Suzuki T., Iwasaki R., Morita K., Hosogane N., Matsuo K., Toyama Y., Suda T. (2007). Induction of DC-STAMP by alternative activation and downstream signaling mechanisms. J. Bone Miner. Res..

[24] Kim K., Lee S.H., Ha Kim J., Choi Y., Kim N. (2008). NFATc1 induces osteoclast fusion via up-regulation of Atp6v0d2 and the dendritic cell-specific transmembrane protein (DC-STAMP). Mol. Endocrinol..

[25] Lu P.J., Zhou X.Z., Liou Y.C., Noel J.P., Lu K.P. (2002). Critical Role of WW Domain Phosphorylation in Regulating Phosphoserine Binding Activity and Pin1 Function. J. Biol. Chem..

[26] Patel S., Mathonet P., Jaulent A.M., Ullman C.G. (2013). Selection of a high-affinity WW domain against the extracellular region of VEGF receptor isoform-2 from a combinatorial library using CIS display. Protein Eng. Des. Sel..

[27] Barman A., Hamelberg D. (2016). Coupled Dynamics and Entropic Contribution to the Allosteric Mechanism of Pin1. J. Phys. Chem. B.

[28] Kim K., Kim J.H., Lee J., Jin H.M., Lee S.H., Fisher D.E., Kook H., Kim K.K., Choi Y., Kim N. (2005). Nuclear factor of activated T cells c1 induces osteoclast-associated receptor gene expression during tumor necrosis factor-related activation-induced cytokine-mediated osteoclastogenesis. J. Biol. Chem..

[29] Ghosh A., Saminathan H., Kanthasamy A., Anantharam V., Jin H., Sondarva G., Harischandra D.S., Qian Z., Rana A., Kanthasamy A.G. (2013). The peptidyl-prolyl isomerase Pin1 up-regulation and proapoptotic function in dopaminergic neurons: Relevance to the pathogenesis of Parkinson disease. J. Biol. Chem..

[30] Florencio-Silva R., Sasso G.R., Sasso-Cerri E., Simões M.J., Cerri P.S. (2015). Biology of Bone Tissue: Structure, Function, and Factors That Influence Bone Cells. Biomed. Res. Int..

[31] Feng X., McDonald J.M. (2011). Disorders of bone remodeling. Annu. Rev. Pathol..

[32] Lewiecki E.M. (2010). Bisphosphonates for the treatment of osteoporosis: Insights for clinicians. Ther. Adv. Chronic Dis..

[33] Milat F., Ebeling P.R. (2016). Osteoporosis treatment: A missed opportunity. Med. J. Aust..

[34] Besaratinia A., Pfeifer G.P. (2004). Genotoxicity of acrylamide and glycidamide. J. Natl. Cancer Inst..

[35] Koyama N., Yasui M., Oda Y., Suzuki S., Satoh T., Suzuki T., Matsuda T., Masuda S., Kinae N., Honma M. (2011). Genotoxicity of acrylamide in vitro: Acrylamide is not metabolically activated in standard in vitro systems. Environ. Mol. Mutagen..

[36] Lee S.H., Choi Y.H., Kim Y.J., Choi H.S., Yeo C.Y., Lee K.Y. (2013). Prolyl isomerase Pin1 enhances osteoblast differentiation through Runx2 regulation. FEBS Lett..

[37] Kanemoto S., Kobayashi Y., Yamashita T., Miyamoto T., Cui M., Asada R., Cui X., Hino K., Kaneko M., Takai T. (2015). Luman is involved in osteoclastogenesis through the regulation of DC-STAMP expression, stability and localization. J. Cell Sci..

[38] Bae W.J., Park J.S., Kang S.K., Kwon I.K., Kim E.C. (2018). Effects of Melatonin and Its Underlying Mechanism on Ethanol-Stimulated Senescence and Osteoclastic Differentiation in Human Periodontal Ligament Cells and Cementoblasts. Int. J. Mol. Sci..

[39] Xu G.G., Zhang Y., Mercedes-Camacho A.Y., Etzkorn F.A. (2011). A reduced-amide inhibitor of Pin1 binds in a conformation resembling a twisted-amide transition state. Biochemistry.

[40] Zhang M., Wang X.J., Chen X., Bowman M.E., Luo Y., Noel J.P., Ellington A.D., Etzkorn F.A., Zhang Y. (2012). Structural and kinetic analysis of prolyl-isomerization/phosphorylation cross-talk in the CTD code. ACS Chem. Biol..

[41] Elokely K.M., Doerksen R.J. (2013). Docking challenge: Protein sampling and molecular docking performance. J. Chem. Inf. Model..

[42] Lionta E., Spyrou G., Vassilatis D.K., Cournia Z. (2014). Structure-based virtual screening for drug discovery: Principles, applications and recent advances. Curr. Top. Med. Chem..

